# Visiting medical student elective and clerkship programs: a survey of US and Puerto Rico allopathic medical schools

**DOI:** 10.1186/1472-6920-10-41

**Published:** 2010-06-07

**Authors:** Paul S Mueller, Linda L McConahey, Laura J Orvidas, Mark C Lee, Juan M Bowen, Thomas J Beckman, Mary J Kasten

**Affiliations:** 1 Division of General Internal Medicine, Mayo Clinic, 200 First Street SW, Rochester, Minnesota, 55905, USA; 2 Mayo School of Graduate Medical Education, 200 First Street SW, Rochester, Minnesota, 55905, USA; 3 Department of Otorhinolaryngology, Mayo Clinic, 200 First Street SW, Rochester, Minnesota, 55905, USA; 4 Division of Primary Care Internal Medicine, Mayo Clinic, 200 First Street SW, Rochester, Minnesota, 55905, USA

## Abstract

**Background:**

No published reports of studies have provided aggregate data on visiting medical student (VMS) programs at allopathic medical schools.

**Methods:**

During 2006, a paper survey was mailed to all 129 allopathic medical schools in the United States and Puerto Rico using a list obtained from the Association of American Medical Colleges. Contents of the survey items were based on existing literature and expert opinion and addressed various topics related to VMS programs, including organizational aspects, program objectives, and practical issues. Responses to the survey items were yes-or-no, multiple-choice, fill-in-the-blank, and free-text responses. Data related to the survey responses were summarized using descriptive statistics.

**Results:**

Representatives of 76 schools (59%) responded to the survey. Of these, 73 (96%) reported their schools had VMS programs. The most common reason for having a VMS program was "recruitment for residency programs" (90%). "Desire to do a residency at our institution" was ranked as the leading reason visiting medical students choose to do electives or clerkships. In descending order, the most popular rotations were in internal medicine, orthopedic surgery, emergency medicine, and pediatrics. All VMS programs allowed fourth-year medical students, and approximately half (58%) allowed international medical students. The most common eligibility requirements were documentation of immunizations (92%), previous clinical experience (85%), and successful completion of United States Medical Licensing Examination Step 1 (51%). Of the programs that required clinical experience, 82% required 33 weeks or more. Most institutions (96%) gave priority for electives and clerkships to their own students over visiting students, and a majority (78%) reported that visiting students were evaluated no differently than their own students. During academic year 2006-2007, the number of new resident physicians who were former visiting medical students ranged widely among the responding institutions (range, 0-76).

**Conclusions:**

Medical schools' leading reason for having VMS programs is recruitment into residency programs and the most commonly cited reason students participate in these programs is to secure residency positions. However, further research is needed regarding factors that determine the effectiveness of VMS programs in residency program recruitment and the development of more universal standards for VMS eligibility requirements and assessment.

## Background

Fourth-year medical students at US and Puerto Rico medical schools commonly participate in clinical electives at institutions other than their own. These visiting medical student (VMS) experiences go by various labels such as visiting medical student electives and clerkships, externships, and audition electives [1-6]. Common wisdom suggests that the motivation of medical students seeking clinical rotations at other institutions is to secure residency positions, and books regarding the residency selection process encourage medical student participation in visiting clerkships to enhance chances for matching at residency programs of choice [7,8]. However, surprisingly little is known about VMS programs at US medical schools. Limited information can be obtained from individual VMS program Web sites and the Association of American Medical Colleges (AAMC) "Extramural Electives Compendium" Web site [9]. However, we are unaware of existing studies that provide aggregate data on VMS programs. Therefore, to better understand the prevalence and characteristics of these programs and the medical students who participate in them, we conducted a survey of allopathic medical schools in the United States and Puerto Rico.

## Methods

Using a mailing list obtained from the AAMC, we developed a paper survey (Additional file 1) that was mailed, along with a postage-paid, self-addressed return envelope, during September 2006 to all 129 allopathic medical schools in the United States and Puerto Rico. The AAMC mailing list provided names of contact persons for each medical school. The survey was mailed to each medical school in care of these contact persons. Each survey was accompanied by a cover letter that instructed the contact person either to complete the survey or to delegate this responsibility to an appropriate individual (eg, VMS program coordinator). The contact person was also instructed to return the survey if the medical school did not have a VMS program. For the purposes of this study, a VMS program was defined as the entity within a medical school that administrates the hosting of medical students from other institutions for clerkship or elective experiences.

The survey item content was based on existing literature and expert opinion (see below). Item response formats were yes-or-no, multiple-choice, fill-in-the-blank, and free-text responses. The survey questions addressed various topics related to VMS programs, including organizational aspects, program objectives, and practical issues pertaining to running these programs. Survey content validity [10] was determined by reviewing themes in the literature regarding VMS programs [1-9,11] and by soliciting input from authors of this study (PSM, LLM, LJO, MCL, JMB, TJB, and MJK) who collectively have more than 120 person-years of experience in medical education and more than 60 person-years of experience in administering a large VMS program [12]. Furthermore, survey content was based on an iterative approach among the author-experts, whereby an initial list of survey questions was submitted by all members, and questions were added or deleted based on consensus reached through ongoing discussions over a period of 3 months. Nonresponding medical schools were recontacted and encouraged to complete the survey. Data collection concluded in February 2007, followed by analysis of the answers to the survey questions.

Survey results were summarized using standard descriptive statistics. Additionally, the characteristics of responding medical schools and nonresponding schools were compared according to 3 characteristics: 1) listing of a VMS program in the AAMC's "Extramural Electives Compendium" Web site [9]; 2) 2007 medical student enrollments [13]; and 3) 2007 *US News & World Report *rankings of medical schools [14]. *US News & World Report *was selected as a basis for comparing responders and nonresponders because this variable is a widely recognized marker of medical school quality and reputation. Therefore, if responders and nonresponders are found to represent medical schools that do not differ greatly in terms of their *US News & World Report *rankings, then this similarity would suggest that they are less likely to differ regarding their dutifulness and transparency in reporting data about visiting medical students. Comparisons for having the VMS program listed in the AAMC's "Extramural Electives Compendium" Web site (yes, no) were made using the Fisher exact test. Comparisons for 2007 enrollments and 2007 *US News & World Report *rankings were determined using the 2-group *t *test. This study was deemed exempt by the Mayo Clinic Institutional Review Board as an education-related study under 45 CFR 46.101(b)4.

## Results

Complete survey response data are presented in Additional file 1. Representatives of 76 medical schools (59%) responded to the survey. Of these, 73 respondents (96%) reported that their medical schools had VMS programs. (These 73 individuals' survey responses comprise the data set for this study. Therefore, unless otherwise indicated, all forthcoming percentages are based on a denominator of 73.) Notably, there were no significant differences between responding schools and nonresponding schools regarding the percentage that had their VMS program listed in the AAMC's "Extramural Electives Compendium" Web site (*P *= .51), 2007 medical student enrollments (*P *= .37), or 2007 *US News & World Report *rankings (*P *= .14). For the comparison of the 2007 *US News & World Report *rankings, a sample size calculation revealed that the numbers of responders and nonresponders provided 80% power to detect an effect size of 0.5, which normally is a small difference. Following is a summary of the survey findings organized by specific themes.

### Organizational aspects of VMS programs

Sixty-one (84%) respondents provided the name of their VMS programs (12 [16%] respondents left the survey item blank). Almost all the reported program names included the terms "visiting," "student," and "program," whereas the terms "elective," "externship," "exchange," and "off-campus" were uncommon. Sixty-one respondents (84%) reported that a single individual or office coordinated all VMS electives and clerkships at their institutions. The most common reason cited for having a VMS program was "recruitment for residency programs" (90%); other reasons are listed in Table 1.

**Table 1:** Reasons cited by US and Puerto Rico allopathic medical schools for having visiting medical student elective and clerkship programs (n = 73)^a^.

Reason	No. (%) of Responses
Recruitment for residency programs	66 (90)
Consistent with education mission of the institution	57 (78)
Enhancement of reputation	28 (38)
Providing teaching opportunities for faculty	12 (16)
Other	11 (15)
Income	6 (8)
Patient referrals	1 (1)

^a ^Responses to question 4 in Additional file 1.

Across the respondents' medical schools, the median number of visiting medical students during academic year 2005-2006 was 96 (range, 0 1,400). During the same year, the median number of medical students enrolled at the respondents' schools was 550 (range, 60-1,168).

Medical schools advertise their VMS programs by using a Web site (71%), word of mouth (49%), other means such as a booth at the American Medical Student Association annual meeting (10%), and direct mailing and advertisements in medical journals (4% each). Of these, using a Web site and word of mouth were cited as the most effective means of advertisement.

### Applying for VMS electives and clerkships

Fifty-nine respondents (81%) reported that applicants were required to use a paper application form, which was mailed to the school, whereas only 6 (8%) required online applications. Five respondents (7%) reported other means, including downloading and printing an application from the school's Web site, which was then mailed to the school. Three (4%) respondents left the survey item blank.

### Eligibility requirements for VMS electives and clerkships

The survey respondents were asked to describe the eligibility requirements for a potential visiting student to participate in electives or clerkships at their institutions. None of the respondents reported allowing first- or second-year medical students, 7 respondents (10%) reported allowing third-year medical students, and 71 respondents (97%) reported allowing fourth-year medical students (2 respondents left the item blank). Sixty-two respondents (85%) reported that osteopathic school students were allowed to do visiting electives or clerkships at their institutions.

The survey respondents also reported a number of additional eligibility requirements, the most common being documentation of immunizations (92%) and previous clinical experience (85%) (Table 2). Of the 62 programs that required clinical experience, 51 (82%) required 33 weeks or more.

**Table 2:** Eligibility requirements for visiting medical student elective and clerkship programs at US and Puerto Rico allopathic medical schools (n = 73)^a^.

Requirement	No. (%) of Responses
Documentation of immunizations	67 (92)
Clinical experience, wk	62 (85)
<16	1 (2)
16-32	5 (7)
33-48	38 (52)
>48	13 (18)
Left item blank	5 (7)
Successful completion of the USMLE Step 1	37 (51)
Letter of recommendation	27 (37)
Medical school transcript	26 (36)

Abbreviation: USMLE, United States Medical Licensing Examination.

^a ^Responses to question 16 in Additional file 1.

### International visiting medical students

Of the 73 survey respondents, 42 (58%) reported that international visiting medical students were allowed to do electives or clerkships at their schools. Of these 42 respondents, 38 (90%) reported that international visiting students must be fluent in English to be eligible for electives or clerkships at their schools and 19 (45%) reported that successful completion of the Test of English as a Foreign Language (TOEFL) was required.

Notably, of the 42 respondents who reported that international visiting medical students were allowed to do electives or clerkships at their schools, 27 (64%) reported that international students comprised 25% or less of the total visiting medical students at their schools, whereas 10 (24%) reported they comprised 26% to 50%, and 1 (2%) reported they comprised 51% or more. Respondents reported hosting international visiting medical students from every region of the world during the 5 years preceding the survey, as follows: Europe, 36 (86%); Canada, 26 (62%); Asia, 21 (50%); Australia and New Zealand, 16 (38%); Africa, 16 (38%); South America, 15 (36%); and Central America, 12 (29%).

### Duration of VMS electives and clerkships

Sixty-three respondents (86%) reported that the length of a single elective or clerkship at their schools for a visiting student was 4 to 5 weeks. At 29 schools responding (40%), the minimum amount of time that a visiting student must spend doing a clerkship or elective at the school was less than 4 weeks, whereas at 39 schools (53%), the minimum amount of time was 4 to 5 weeks. At 35 schools (49%), the maximum amount of time was 6 to 8 weeks, whereas 34 schools (47%) listed a maximum time of 7 to 8 weeks (5 respondents [7%] listed "no limit"). Despite these varying time requirements and allowances, at 60 schools (82%), the average amount of time a visiting student actually spent at the host institution was 4 to 5 weeks.

### Elective and clerkship prioritization and choices

Seventy survey respondents (96%) reported that, for a given elective or clerkship, medical students at their own institutions were given priority over visiting students. Nevertheless, 57 respondents (78%) reported that visiting students were allowed to do electives and clerkships in all departments and divisions at their institutions. Sixty-six respondents (90%) listed the 3 most popular electives and clerkships for visiting medical students at their institutions (7 [10%] respondents left the survey item blank). In descending order, the most popular rotations were in general and subspecialty internal medicine, orthopedic surgery, emergency medicine, general and subspecialty pediatrics, and obstetrics and gynecology; other specialties were listed only occasionally.

### Reported reasons medical students do visiting electives and clerkships

The survey respondents were asked to rank reasons visiting medical students choose to do electives or clerkships at their institutions. Among various choices, "desire to do a residency at our institution" (47%) and "reputation of our institution" (22%) were ranked first most often (Table 3). Notably, only 1 respondent (1%) reported that visiting students were guaranteed an interview for a future residency program position at their institution.

**Table 3:** Top reason cited by survey respondents why visiting medical students do clerkships or electives at US and Puerto Rico allopathic medical schools with visiting medical student programs (n = 73)^a^.

Reason	No. (%) of Responses
Desire to do a residency at our institution	34 (47)
Reputation of our institution	16 (22)
Location of our institution	7 (10)
Learning opportunities at our institution	4 (5)
Word-of-mouth encouragement	3 (4)
Cost	2 (3)
Other reasons	2 (3)
Left item blank	5 (7)

^a ^Responses to question 30 in Additional file 1.

### Evaluation of visiting medical students

Of the 73 survey respondents, 57 (78%) reported that visiting students were not evaluated any differently than their own medical students. Sixty-six (90%) reported that grades were part of the evaluation of visiting students; the following grading systems were reported: 24 (36%) reported an "honors, high pass, pass, marginal pass, fail" system; 14 (21%) reported a "pass/fail" system; 8 (12%) reported letter grades (A, B, C, etc); and 20 (30%) reported "other" (various grading systems depending on the elective or clerkship).

Thirty-eight respondents (52%) reported that they completed evaluation forms provided by the students' home medical schools, 23 (32%) that they completed their own evaluation forms and forms provided by the students' home schools, 4 (5%) that they completed only their own evaluation forms, 3 (4%) reported "other," and 5 (7%) did not respond to the survey question (left the item blank). Fifty respondents (68%) reported that the specific elective or clerkship director completed evaluation forms for visiting students, whereas 3 (4%) reported that the VMS program coordinator at their schools completed the forms; 17 (23%) reported "other." These "other" individuals, according to free-text entries, included "preceptor," "attending physician," and "residents." Three (4%) respondents did not respond to the survey question.

Forty-six respondents (63%) reported that the visiting student's home medical school received the evaluation. Fourteen respondents (19%) reported the visiting student and the student's home medical school received the evaluation, 6 (8%) reported only the visiting student received the evaluation, and 3 respondents (4%) reported "other." Three respondents (4%) reported that records on visiting students were kept for less than 1 year, 26 (36%) reported 1 to 3 years, 14 (19%) reported 4 to 7 years, 2 (3%) reported more than 7 years, and 22 (30%) reported "indefinitely."

### Challenges related to having and maintaining a VMS program

All the survey respondents reported challenges related to having and maintaining a VMS program. The most commonly cited challenges were insufficient overall slots to meet the demand (36%), insufficient slots to meet the demand for a specific specialty (26%), and widely variable eligibility criteria among departments and divisions (14%) (Table 4).

**Table 4:** Top challenge cited by survey respondents related to having and maintaining a visiting medical student program at US and Puerto Rico allopathic medical schools (n = 73)^a^.

Challenge	No. (%) of Responses
Overall, insufficient elective or clinical rotation slots to meet the demand	26 (36)
Insufficient elective or clinical rotation slots to meet the demand for a specific specialty	19 (26)
Widely variable eligibility criteria among departments and divisions at our institution	10 (14)
Insufficient funds	6 (8)
Lack of underrepresented minority visiting students	5 (7)
Lack of qualified visiting students	4 (5)
No response	3 (4)

^a ^Responses to question 29 in Additional file 1.

### Success of former visiting medical students in securing a residency position

The survey respondents were asked to report the number of their new first-year residents who were former visiting medical students at their institutions. Across the respondents' medical schools, the median number of new first-year resident physicians during academic year 2006-2007 who were former visiting medical students at the respondents' schools was 6 (range, 0-76).

## Discussion

To our knowledge, this is the first study to provide aggregate data regarding the characteristics of VMS programs and participating students at US and Puerto Rico medical schools. Important findings were that most medical schools have VMS programs, the most popular rotations are in internal medicine, demand outweighs available positions, and eligibility and evaluation standards are inconsistent within and between institutions. About half the programs allow international students; this result is consistent with data on the AAMC's "Extramural Electives Compendium" Web site, which lists 117 US medical schools with VMS programs, of which 47 (40%) allow international medical students to do visiting electives and clerkships at their institutions [9]. (Notably, the AAMC's "Extramural Electives Compendium" Web site does not present data regarding VMS programs in aggregate; these data were abstracted manually and compiled by one of us [PSM].)

The most commonly reported reason for having a VMS program is to recruit residents. This finding may help explain previous research showing that approximately half of all fourth-year US medical students participate in electives and clerkships at institutions other than their own medical schools [1-5] and also supports advice that medical students should pursue visiting electives and clerkships to enhance their chances of securing residency positions at the host institution [7,8]. In fact, most visiting medical students are in their fourth year and therefore are likely contemplating their residency training options. Indeed, the survey respondents ranked "desire to do a residency at our institution" as the most important reason visiting medical students choose to do electives or clerkships.

A majority of VMS programs require substantial clinical experience. Consequently, most VMS programs are limited to third- or fourth-year students. In addition, most VMS programs require fluency in English, documentation of immunizations, and completion of United States Medical Licensing Examination (USMLE) Step 1; some have additional requirements. These findings suggest that VMS programs make an effort to ensure that visiting students have skills and educational levels comparable to their own students, which enhances the visiting clerkship experience for the students, faculty, and patients. Another possibility is that VMS programs "screen" prospective visiting students to accommodate only those who might be competitive for residency programs.

Nonetheless, VMS programs may not recruit students as effectively as they hope to. Only 1 VMS program guaranteed interviews to visiting medical students. Furthermore, we found substantial variability in the effectiveness of VMS programs at recruiting residents and former visiting medical students who make up first-year residency classes at the respondents' institutions ranged widely (range, 0-76). Moreover, a previous single-institution study showed that students who participated in VMS programs were not more successful in obtaining their first choice of residency than students who did not participate in VMS programs [11].

At the time of our survey, more than 80% of VMS programs required paper applications and less than 10% required online applications. Since then--during April 2008--the AAMC launched the Visiting Student Application Service (VSAS), which is an online application service for VMS programs at US medical schools. To date, 63 US medical schools with VMS programs use the VSAS. This system, however, does not allow students to enter information related to clinical experiences, upload letters of recommendation, or enter USMLE Step 1 scores. In addition, potential international visiting medical students cannot use the VSAS [15]. Our survey findings suggest that updating the VSAS to allow it to upload the aforementioned information would be desirable to most VMS programs.

A majority of respondents reported that visiting medical students were evaluated no differently than their own medical students. Only 5% of programs reported using only their own evaluation forms for visiting medical students, 52% of programs reported using only the students' home institutions' evaluation forms, and 32% reported using both. It is likely, however, that medical schools evaluate students similarly (eg, knowledge, presentations skills, professionalism) and that overlap exists among the schools' evaluation forms. Nevertheless, in our experience administering a VMS program that accommodates about 350 visiting students per year [12], completing home institutions' evaluation forms can be logistically complicated. Given the large number of medical schools that have VMS programs and the large number of visiting medical students who participate in them, a standardized evaluation form for visiting medical students might be desirable.

All the respondents reported challenges related to maintaining a VMS program. One common challenge was insufficient numbers of elective and clerkship slots, possibly because most institutions give their own medical students priority for electives and clerkships. In addition, accommodating visiting students strains educational resources, including faculty availability. Furthermore, some electives and clerkships are more popular than others. Respondents to our survey listed general internal medicine and internal medicine subspecialty clerkships and electives, orthopedic surgery, and emergency medicine as the most popular among visiting students. Therefore, VMS programs might consider ways to expand availability for these electives and clerkships and determine reasons why some specialties are not targeted by visiting students.

Our study has a number of limitations. First, only 59% of medical schools responded to the survey and nonrespondents may have had unique perspectives on their VMS programs that would have influenced the results. However, there were no significant differences between the responding and nonresponding schools regarding the percentages of VMS programs listed in the AAMC's "Extramural Electives Compendium" Web site, 2007 medical student enrollments, or 2007 *US News & World Report *rankings, thereby adding validity to the results. Second, we examined the perspectives of VMS programs, not the perspectives of actual visiting medical students. The survey respondents' impressions that medical students participate in VMS programs primarily to secure residency positions should be confirmed in future studies by surveying actual visiting medical students. Other reasons medical students participate in VMS programs might include evaluating an institution or residency program and comparing it to others, enhancing knowledge and skills, observing alternative practice styles and systems, and the enjoyment of travel [11]. It is also possible that US and international medical students participate in VMS programs for different reasons. All these medical student perspectives should be further explored.

Finally, our survey did not assess variables that predict success among visiting medical students, which would be especially informative to medical schools with VMS programs that have limited clerkship slots and other resources. Furthermore, although a prior single-institution study [11] showed that students who participated in visiting electives and clerkships were not more successful in obtaining their first choice of residency position than students who did not, schools nevertheless claim that the primary reason for having a VMS program is to enhance residency program recruitment. Thus, the relationship between participating in VMS programs and securing residency positions needs further study.

## Conclusions

We report the first study on the characteristics of VMS programs and participating students at US and Puerto Rico medical schools. We found that most medical schools have VMS programs, the most popular rotations among visiting students are in general and subspecialty internal medicine, and eligibility and evaluation standards are inconsistent. Many medical schools view VMS programs as useful for recruiting medical students into residency programs. However, first-year residency classes at some institutions comprise only a few previous visiting medical students. Therefore, further research is needed regarding development of more uniform eligibility and evaluation criteria for visiting medical students and factors that determine the effectiveness of VMS programs in residency program recruitment.

## Abbreviations

AAMC: Association of American Medical Colleges; TOEFL: Test of English as a Foreign Language; USMLE: United States Medical Licensing Examination; VMS: visiting medical student (program); VSAS: Visiting Student Application Service.

## Competing interests

Dr Mueller has a consulting relationship with Boston Scientific (Patient Safety Advisory Board) and is an Associate Editor for *Journal Watch*. Neither relationship is directly related to the content of this paper. The other authors have no competing interests to report.

## Authors' contributions

PSM conceived and designed the study, participated in collection, coordination, and analysis of data, and writing of the manuscript. LLM conceived and designed the study, participated in collection, coordination, and analysis of data, and writing of the manuscript. LJO conceived and designed the study, participated in data analysis, and writing of the manuscript. MCL conceived and designed the study, participated in data analysis, and writing of the manuscript. JMB conceived and designed the study, participated in data analysis, and writing of the manuscript. TJB participated in data analysis and writing of the manuscript. MJK conceived and designed the study, participated in data analysis, and writing of the manuscript. All authors read and approved the final manuscript.

## Pre-publication history

The pre-publication history for this paper can be accessed here:

http://www.biomedcentral.com/1472-6920/10/41/prepub

## Supplementary Material

Additional file 1 **Survey questions and responses (answers to these questions comprised the data set for this study)**. Click here for file
