# Supplementary material for: Visiting medical student elective and clerkship programs: a survey of US and Puerto Rico allopathic medical schools
Source: BMC Med Educ. 2010 Jun 7;10:41. doi: 10.1186/1472-6920-10-41 (PMC2893187; doi:10.1186/1472-6920-10-41)
Supplement: Additional file 1 — Survey questions and responses (answers to these questions comprised the data set for this study). [file 1472-6920-10-41-S1.DOC]

**Additional file 1.** Visiting medical student elective and clerkship programs: a survey of US and Puerto Rico allopathic medical schools: survey questions and responses (answers to these questions comprised the data set for this study).

1. Does your school have a program for visiting medical students? (yes/no)

Yes 73 (96%)

No 3 (4%)

1. What do you call your program for visiting medical students who do electives or rotations at your institution?

N = 73

Respondents 61 (84%)

Responses:

“Visiting Student Program” 30

“Visiting Student Elective Program” 10

“Visiting Medical Student Program” 8

“Senior Elective Program” 3

“Visiting Senior Elective Program” 1

“Visiting Elective Program” 1

“The Externship Program” 1

“Off-Campus Student Elective Program” 1

“Guest Student Program” 1

“Exchange Student Program” 1

“Exchange Clerk Program” 1

“Electives at [name of institution]” 1

“Clinical Elective for Visiting Students” 1

“4^th^ Year Visiting Student Elective Program” 1

1. Does a single individual or office coordinate all of the visiting medical students who do electives or rotations at your institution? (yes/no)

N=73

Yes 61 (84%)

No 10 (14%)

Left item blank 2 (3%)

1. Why do you have a program for visiting medical students who do electives or rotations at your institution? (check all that apply):

N=73

Recruitment for residency programs 66 (90%)

Income 6 (8%)

Patient referrals 1 (1%)

Enhancement of reputation 28 (38%)

Provide teaching opportunities 12 (16%)

Consistent with education mission 57 (78%)

Other^*^ 11 (15%)

*Other comments:

“Cross fertilization”

“Provide our students exposure to people/students from other countries and their medical education”

“To provide students an opportunity to learn elsewhere”

“Provide learning opportunities for our students”

“Because our students go elsewhere for electives—reciprocal”

“To help ensure our students are able to go to other schools”

“Fairness—our students rotate away”

“To establish relationship with other schools—we want them to accept our students during their away rotations”

(2 respondents checked “other” but did not comment)

1. How many medical students were enrolled at your institution during 2005?

N=73

Respondents 61

Left item blank 12

Median 560

Range 60-1,168

1. During 2005, how many visiting medical students did electives or rotations at your institution?

N=73

Respondents 69

Left item blank 4

Median 96

Range 0-1,400

1. Of the new resident physicians who matriculated at your institution during 2006, how many were former visiting medical students at your institution?

N=73

Respondents 23

Left item blank 50

Median 6

Range 0-76

1. What is the typical length of a single elective or rotation for visiting medical students at your institution?

N=73

<2 weeks 0 (0%)

2-3 weeks 2 (3%)

4-5 weeks 63 (86%)

6 weeks 0 (0%)

7-8 weeks 1 (1%)

Other 5 (7%)

Left item blank 2 (3%)

1. What is the minimum amount of time that a visiting medical student must spend doing an elective or rotation at your institution?

N=73

<2 weeks 1 (1%)

2-3 weeks 28 (38%)

4-5 weeks 39 (53%)

6 weeks 0 (0%)

7-8 weeks 0 (0%)

Other 4 (5%)

Left item blank 1 (1%)

1. What is the maximum amount of time that a visiting medical student is allowed to spend doing electives or rotations at your institution?

N=73

<2 weeks 0 (0%)

2-3 weeks 0 (0%)

4-5 weeks 3 (4%)

6 weeks 1 (1%)

7-8 weeks 34 (47%)

No limit 5 (7%)

Other^*^ 29 (40%)

Left item blank 1 (1%)

^*^ Almost all the respondents who answered “Other” wrote “12 weeks” or “3 months” in the comments section.

1. What is the average amount of time that a visiting medical student spends doing an elective or rotation at your institution?

N=73

<2 weeks 0 (0%)

2-3 weeks 1 (1%)

4-5 weeks 60 (82%)

6 weeks 1 (1%)

7-8 weeks 4 (6%)

Other 6 (8%)

Left item blank 1 (1%)

1. What are the 3 most popular electives or rotations for visiting medical students doing electives or rotations at your institution?

N = 73

Respondents 66 (90%)

Responses (each row represents a response from a different program):

“No consistently chosen electives”

“Orthopedic Surgery, Internal Medicine, Urology”

“Emergency Medicine, Physical Medicine and Rehabilitation, Orthopedic Surgery”

“Plastic Surgery, Orthopedic Surgery, Neurosurgery”

“Internal Medicine, Obstetrics and Gynecology, Family Medicine”

“Obstetrics and Gynecology, Family Medicine, Pediatrics”

“Emergency Medicine, Radiology, Pediatrics”

“Medicine, Orthopedic Surgery, Pediatrics”

“Emergency Medicine, Pediatrics, Surgery”

“Emergency Medicine, Dermatology, Cardiology”

“Dermatology, Anesthesia, Ophthalmology”

“Pediatric Infectious Diseases, Pediatric Gastroenterology, Pediatrics”

“Cardiology, General Surgery”

“Emergency Medicine, Radiology, Surgery”

“Orthopedic Surgery, Anesthesiology, Emergency Medicine”

“Emergency Medicine, Dermatology, Urology”

“Orthopedic Surgery, Cardiology, Endocrinology”

“Varies from year to year”

“Internal Medicine, Anesthesia, Orthopedic Surgery”

“Cardiology, Emergency Medicine”

“Emergency Medicine, Pediatrics, Dermatology”

“Adult Emergency Medicine, Dermatology, Anesthesiology”

“Emergency Medicine, Orthopedics, Pediatrics”

“Family Medicine, Anesthesia, Orthopedics”

“Radiology, Orthopedic Surgery, Neurology”

“Emergency Medicine, Dermatology, Orthopedic Surgery”

“Anesthesiology, Obstetrics and Gynecology, Orthopedic Surgery”

“Surgery, Family Practice”

“Dermatology, Orthopedic Surgery, Emergency Medicine”

“Electives in Internal Medicine and Pediatrics”

“General Medicine, Pediatric Infectious Disease, Cardiology”

“Surgery, Orthopedic Surgery, Pediatric Infectious Disease”

“Emergency Medicine, Pediatrics, Internal Medicine”

“Gastroenterology, Inpatient Internal Medicine, Rheumatology”

“Surgery, Dermatology”

“Dermatology, Anesthesiology, Cardiology”

“Maternal and Child Health, Gynecologic Surgery, Family Medicine”

“Anesthesiology, Orthopedic Surgery, Emergency Medicine”

“Internal Medicine”

“Pediatrics, Surgery, Internal Medicine”

“Orthopedic Surgery, Internal Medicine, Radiology”

“Surgery, Internal Medicine, Emergency Medicine”

“Cardiology, Orthopedic Surgery, Emergency Medicine”

“Medicine, Pediatrics, Cardiology”

“Family Medicine, Nephrology, Obstetrics and Gynecology”

“Emergency Medicine, Internal Medicine, Surgery”

“Radiology, High-Risk Obstetrics, Nephrology”

“Pediatrics, Internal Medicine, Anesthesiology”

“Advanced Surgery, Orthopedics, Anesthesiology”

“Orthopedic Surgery, Emergency Medicine, Plastic Surgery”

“Pediatrics, Internal Medicine, Surgery”

“Emergency Medicine, Pediatric Gastroenterology, Radiology”

“Internal Medicine, Pediatrics, Orthopedic Surgery”

“Orthopedic Surgery”

“Orthopedic Surgery, Otolaryngology, Ophthalmology”

“Emergency Medicine, Anesthesia, Radiology”

“Orthopedic Surgery, Ophthalmology, Anesthesiology”

“Anesthesiology, Physical Medicine and Rehabilitation, Ophthalmology”

“No pattern”

“Orthopedic Surgery, Internal Medicine, Pediatrics”

“Orthopedic Surgery, Obstetrics and Gynecology, Emergency Medicine”

“Emergency Medicine, Pediatrics, Anesthesiology”

“Radiology, Orthopedic Surgery, Anesthesiology”

“Anesthesiology, Emergency Medicine”

“Emergency Medicine”

“Internal Medicine, Orthopedic Surgery, Family Medicine”

1. For a given clerkship elective or rotation, do you give priority to your own medical students over visiting students? (yes/no)

N=73

Yes 70 (96%)

No 2 (3%)

Left item blank 1 (1%)

1. How do potential visiting medical students apply to do electives or rotations at your institution?

N=73

Online application 6 (8%)

Paper mailed application 59 (81%)

Other^*^ 5 (7%)

Left item blank 3 (4%)

*Other comments:

“Combination—online application, but printed out and verified by school”

“Paper application w/supporting documents can be mailed or faxed”

“The application is online but gets printed and mailed to us”

“E-mail application”

“Download application from Web site and mail in”

1. Which of the following students do you allow to do visiting electives or rotations at your institution? (check all that apply)

N=73

First-year medical students 0 (0%)

Second-year medical students 0 (0%)

Third-year medical students 7 (10%)

Fourth-year medical students 71 (97%)

Left item blank 2 (3%)

1. Which of the following are necessary for visiting students to be eligible to do electives or rotations at your institution? (check all that apply)

N=73

Letter of recommendation 27 (37%)

Medical school transcript 26 (36%)

Completion of USMLE Step 1 37 (51%)

Documentation of immunizations 67 (92%)

Clinical experience 62 (85%)

If clinical experience is required, how much?

n=62

<16 weeks 1 (2%)

16-32 weeks 5 (8%)

33-48 weeks 38 (61%)

More than 48 weeks 13 (21%)

Left item blank 5 (8%)

1. What fees and tuition (in US dollars) do you charge visiting medical students who do electives or rotations at your institution?

Fee No. Median (Range)

Application fee

US students 29 50 (25-125)

International students 22 100 (25-500)

Processing fee

US students 10 50 (25-100)

International students 5 50 (25-500)

Tuition

US students 4 175 (25-300)

International students 8 2,250 (25-3,454)

1. Are international medical students eligible to do electives or rotations as visiting students at your institution? (yes/no)

N=73

Yes 42 (58%)

No 30 (41%)

Left item blank 1 (1%)

If yes, how many international visiting medical students did electives or rotations at your institution during 2005?

N=42

Respondents 28

Left item blank 14

Median 12.5

Range 0-400

If yes, of the visiting medical students who do electives or rotations at your institution, what percentage, on average per year, are international students?

N=42

25% or less 27 (64%)

26% to 50% 10 (24%)

51% or more 1 (2%)

Left item blank 4 (10%)

During the past 5 years, from which of the following geographic areas have your visiting students come? (check all that apply)

N=42

Europe 36 (86%)

Canada 26 (62%)

Asia 21 (50%)

Australia and New Zealand 16 (38%)

Africa 16 (38%)

South America 15 (36%)

Central America 12 (29%)

Do you require fluency in English?

N=42

Yes 38 (90%)

No 3 (7%)

Left item blank 1 (2%)

If yes, do you require successful completion of the Test of English as a Foreign Language (TOEFL)?

N=42

Yes 19 (45%)

No 19 (45%)

Left item blank 4 (10%)

1. Do you allow students from osteopathic (DO) schools to do visiting electives or rotations at your institution? (yes/no)

N=73

Yes 62 (85%)

No 8 (11%)

Left item blank 3 (4%)

1. Are visiting medical students allowed to do electives and rotations in all departments and divisions at your institution? (yes/no)

N=73

Yes 57 (78%)

No 13 (18%)

Left item blank 3 (4%)

1. Are visiting medical students who do electives or rotations at your institution guaranteed an interview for a residency position at your institution? (yes/no)

N=73

Yes 1 (1%)

No 63 (86%)

Left item blank 9 (12%)

1. How do you advertise your visiting medical student program? (check all that apply)

N=73

No advertising 23 (32%)

Web site 52 (71%)

Direct mailing 3 (4%)

Advertisements in medical journals 3 (4%)

Word of mouth 36 (49%)

Other 7 (10%)

Of these options, which do you find to be the most effective? (check one)

N=73

No advertising 9 (12%)

Web site 37 (51%)

Direct mailing 0 (0%)

Advertisements in medical journals 0 (0%)

Word of mouth 16 (22%)

Other 1 (1%)

Left item blank 10 (14%)

1. Are visiting medical students who do electives or rotations at your institution evaluated any differently than your own students? (yes/no)

N=73

Yes 9 (12%)

No 57 (78%)

It varies 6 (8%)

Left item blank 1 (1%)

1. Are grades part of the evaluation of visiting medical students who do electives or rotations at your institution? (yes/no)

N=73

Yes 66 (90%)

No 7 (10%)

If yes, what types of grades are used?

N=66

Pass/fail 14 (21%)

Letter grades [A, B, C, etc] 8 (12%)

Honors, high pass, pass, marginal pass, fail 24 (36%)

Other^*^ 20 (30%)

*Other comments:

“Depends on the form student brings”

“Honors, Pass, or Fail”

“Outstanding, excellent, satisfactory”

“H, HP, P, F”

“Honors, Pass, Fail”

“Depends on school”

“Depends on home institution”

“Depends on individual student’s evaluation”

“Honors/High Pass/Pass/Fail”

“Use student’s form”

“Honors, High Pass, Pass, Low Pass, Incomplete, and Fail”

“Depends on what is required by student’s school”

“Depends on form used”

“Their school’s grading scheme”

“Whatever home school chooses”

“Honors, Letters, Satisfactory, Unsatisfactory”

“Honors, Satisfactory, Unsatisfactory”

“Depending on what the visiting student’s home school requires”

(2 respondents checked “other” but did not comment)

1. By what means are evaluations of visiting medical students who do electives or rotations at your institution completed?

N=73

Our own form 4 (5%)

Form provided by the student’s home institution 38 (52%)

Our own and student’s home institution form 23 (32%)

A summary letter 0 (0%)

Other 3 (4%)

Left item blank 5 (7%)

1. Who completes the evaluations of visiting medical students who do electives or rotations at your institution?

N=73

Clerkship director 50 (68%)

Visiting medical student program coordinator 3 (4%)

Other^*^ 17 (23%)

Left item blank 3 (4%)

*Other comments:

“The individual faculty member who coordinates the elective”

“Each department has a designee”

“Elective sponsor”

“Elective preceptor”

“Faculty preceptors”

“Physician the student follows most closely”

“Physician that the student is assigned to”

“The rounding physician of the student”

“Attending physician”

“Faculty offering elective”

“Faculty preceptor”

“Attending”

“Whoever works with the student”

“Senior residents”

“Attending working with the student”

“Their residents and supervisor”

“Preceptor/faculty”

(3 respondents checked “other” but did not comment)

1. Who receives the evaluation of the visiting medical student who does an elective or rotation at your institution?

N=73

Student 6 (8%)

Student’s medical school 46 (63%)

Both student and the student’s medical school 14 (19%)

Other 3 (4%)

Left item blank 4 (5%)

1. For how long do you keep records on visiting medical students who do electives or rotations at your institution?

N=73

No records kept 2 (3%)

<1 year 3 (4%)

1-3 years 26 (36%)

4-7 years 14 (19%)

7 years 2 (3%)

Indefinitely 22 (30%)

Left item blank 4 (5%)

1. Which of the following are challenges of having a visiting medical student program at your institution? (check all that apply)

N=73

Insufficient funds 6 (8%)

Lack of qualified students 4 (5%)

Lack of underrepresented minority students 5 (7%)

Variable departmental eligibility criteria 10 (14%)

Overall insufficient elective slots to meet demand 26 (36%)

Insufficient specialty elective slots to meet demand 19 (26%)

1. Rank the following reasons why visiting students choose to do electives or rotations at your institution with 1st as the most common reason (only the top 3 choices shown) (N=73).

Reason 1st 2nd 3rd

Location 7 (10%) 9 (12%) 8 (11%)

Reputation 16 (22%) 20 (27%) 8 (11%)

Learning opportunities 4 (5%) 9 (12%) 25 (34%)

Desire for residency* 34 (47%) 13 (18%) 9 (12%)

Cost 2 (3%) 4 (5%) 2 (3%)

Word-of-mouth 3 (4%) 6 (8%) 4 (5%)

Other 2 (3%) 1 (1%) 1 (1%)

*Desire for a residency position at the host institution
